# Effective Dispensing Methods for Loading Drugs Only to the Tip of DNA Microneedles

**DOI:** 10.3390/pharmaceutics12100954

**Published:** 2020-10-10

**Authors:** Moonjeong Bok, Zhi-Jun Zhao, Soon Hyoung Hwang, Hyeok-Joong Kang, Sohee Jeon, Jiwoo Ko, Jiwon Jeong, Young Seok Song, Eunju Lim, Jun-Ho Jeong

**Affiliations:** 1 Nano-Convergence Mechanical Systems Research Division, Korea Institute of Machinery and Materials, Daejeon 34103, Korea; bokmj@kimm.re.kr (M.B.); zhaozhijun@kimm.re.kr (Z.-J.Z.); soon814@kimm.re.kr (S.H.H.); kanghj@kimm.re.kr (H.-J.K.); sjeon@kimm.re.kr (S.J.); jjiwoo@kimm.re.kr (J.K.); 2 Department of Fiber System Engineering, Dankook University, Yongin 448-701, Korea; jiwon6350@gmail.com (J.J.); ysong@dankook.ac.kr (Y.S.S.); 3 Department of Science Education/Creative Convergent Manufacturing Engineering, Dankook University, Yongin 448-701, Korea; 4 Department of Nano Mechatronics, University of Science and Technology (UST), Daejeon 34113, Korea

**Keywords:** microneedles, dispensing method, anti-diffusion, FITC, micro-jetting

## Abstract

Here, we propose a novel and simple method to efficiently capture the diffusion of fluorescein isothiocyanate (FITC)-dextran from a biocompatible substance and load the drug only to the tip of DNA microneedles. A dispensing and suction method was chosen to fabricate the designed microneedles with efficient amounts of FITC as the drug model. Importantly, the vacuum process, which could influence the capturing of FITC diffusion from the tip, was evaluated during the manufacturing process. In addition, the simulations were consistent with the experimental results and showed apparent diffusion. Moreover, dextrans of different molecular weights labeled with FITC were chosen to fabricate the tip of microneedles for demonstrating their applicability. Finally, a micro-jetting system with a micro-nozzle (diameter: 80 μm) was developed to achieve the accurate and rapid loading of small amounts of FITC using the anti-diffusion and micro-jetting methods. Our method not only uses a simple and fast manufacturing process, but also fabricates the tips of microneedles more efficiently with FITC compared with the existing methods. We believe that the proposed method is essential for the clinical applications of the microneedle drug delivery platform.

## 1. Introduction

Microneedles (MNs) are widely used in the cosmetic and medical fields for improving wrinkles [1,2] and for painless drug injection [3,4,5], respectively. In particular, various drug delivery systems [6,7,8,9] with typical MN patches have been developed to deliver drugs more efficiently. Among them, dissolvable MNs, as skin-attached devices, fabricated with biocompatible materials, have garnered attention in the field of drug delivery [10,11,12]. For example, dissolving MNs have also been proposed to treat eye diseases. For eye drops, a large amount of drug is required to achieve an effective local drug concentration owing to structural barriers of the eye, such as the corneal epithelium and blood–retinal barrier [13,14,15]. These large amounts of drugs can increase the risk of off-target side effects. Therefore, MNs have been used as a therapeutic means to overcome this structural barrier. Previous studies have reported a method wherein a drug is embedded on the MNs using various methods, including dipping, coating, and drawing lithography [16,17,18,19,20]. In addition, biocompatible and self-dissolving MNs such as microfluidic channels ensure that the drug is absorbed in the body. To enhance the efficiency of drug delivery using MNs, various patches have been studied using different methods. Recently, Ye et al. [21] fabricated tip-dissolving MNs via the dipping and lifting up method and demonstrated efficient drug delivery. In addition, Chen et al. [22] proposed an adjustable apparatus that can be lifted and lowered to fabricate coated polymer MNs with homogeneous controlled drug loading. Lee et al. [23] presented dissolvable polyvinylpyrrolidone (PVP)-based MNs that do not require needle removal, while achieving the rapid release of encapsulated insulin via a centrifuging method. Moreover, Chen et al. [24] proposed the fabrication of MNs using compressed droplets of magnetic rheological fluid under an external magnetic field to quickly fabricate MNs without a conventional mold and mask. Ning et al. [25] developed a strategy of freeze spray coating, whereby a solution containing coating molecules was rapidly frozen in liquid nitrogen in advance to achieve uniform coating on the MNs, for a double-layer structure. These studies have demonstrated the improved fabrication of MNs and efficient performance for drug delivery. However, the effective drug-loading amount and fabrication should be improved. 

In addition, our group developed a drug delivery system with salmon DNA and hyaluronic acid drug-embedded MNs, and achieved higher drug delivery efficiency by applying physical enhancers, including electricity, ultrasound, and combinations thereof [26,27,28]. However, we found that it is also critical to efficiently control the amount of drugs delivered to the body and avoid unnecessary drugs (like drugs on the patch backplate) [29]. 

To date, studies have focused on developing a drug delivery system and fabricating various patches. Therefore, to achieve more effective drug delivery and absorption, we aimed to develop a novel and simple method to fabricate only the tip of dissolvable DNA MNs with drugs for efficient drug distribution. In addition to the drug contained in the MN, the stability of the material for drug loading is an important factor of the structural material. DNA has the advantages of biocompatibility, excellent mechanical robustness for biofunctional applications, and ability to stably load chemical molecules into DNA [26,27,30]. Chemical drugs often cause adverse effects, such as cytotoxicity, low loading efficiency, and explosive release [30]. Therefore, we applied DNA-based MNs that can stably load chemical drugs.

A simple dispensing and suction method was chosen to accurately fabricate the MN tips with drugs. In addition, we tested drugs of different molecular weights to demonstrate the applicability of our method. In the two-step molding process, as the diffusion of the drug solvent must be considered for effective drug loading, a fluorescence analysis was performed according to the vacuum timing of the DNA solution. Additionally, our simulations showed the apparent diffusion of drugs that may occur during the manufacturing process. Finally, a micro-jetting system with micro-nozzles was applied to load the accurate amount of the drug onto the tip. Therefore, this method improves drug distribution and minimizes drug loss in the MN-based drug delivery system.

## 2. Materials and Methods 

### 2.1. Sample Preparation

#### 2.1.1. Preparation of FITC-Dextran Solution

Fluorescein isothiocyanate (FITC)-labeled dextrans of average molecular weights 10, 70, and 2000 kDa were purchased from Sigma-Aldrich (St. Louis, MO, USA). Dextran solutions (10 mg/10 mL) were prepared by dissolving appropriate amounts of dextran powder in deionized water. 

#### 2.1.2. Preparation of DNA Solution

DNA was dissolved in de-ionized (DI) water to obtain 1.0% wt DNA. A homogeneous mixture of DNA and DI water was obtained by magnetic stirring at approximately 500 rpm for 8 h at room temperature.

#### 2.1.3. FITC-Dextran Loading

##### A. Dispensing and Suction Method

The prepared FITC-dextran solution (1.5 mL; 10, 70, or 2000 kDa) was dispensed into a mold. The solution dispensed in the mold was vacuumed for 5 min, and then 1.2 mL of the solution was sucked with a syringe and dried in an oven at 50 °C for 30 min. After drying, the residual FITC-dextran was stripped off with a tape.

##### B. Micro-Jetting Method

The prepared FITC-dextran solution was jetted using the MicroFab JetDrive system. The micro-jetting system consisted of an orifice of diameter 80 μm (MJ-AT-01; Microfab, Plano, TX, USA) and a reservoir. The drive shape was an anodic trapezoidal waveform. Drop formation and jetting in the mold were analyzed using stroboscopic images captured with a CCD camera (rise time, 25 μs; dwell time, 30 μs; fall time, 4 μs; echo time, 20 μs; and final rise time, 10 μs). To accurately visualize the drop formation behavior, the firing frequency was set as 200 Hz. The droplet number was determined by setting the trigger mode to burst type. 

#### 2.1.4. Fabrication of FITC-Dextran-Loaded DNA-Based MNs 

**Figure 1:**
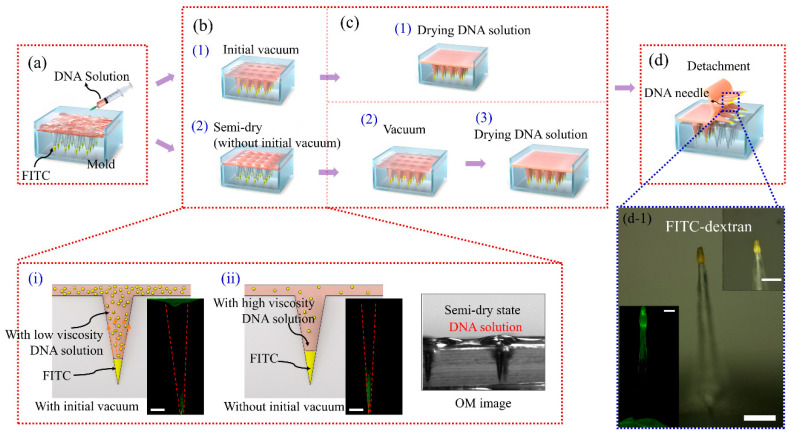
Fabrication process of FITC-loaded DNA microneedle with and without initial vacuum. (**a**) Loading of the 1% wt DNA solution into a mold loaded with FITC. (**b**) With/without initial vacuum step, (1) with initial vacuum, (2) without initial vacuum after pouring, and semi-drying. The enlarged image (i and ii) is the schematic diagram and pictures of the FITC-dextran diffusion process in DNA solution during the needle manufacturing process and optical image of the semi-dry state in DNA solution (scale bar: 200 μm). (**c**) (1) Drying DNA solution, (2) vacuum process, (3) drying DNA solution; (**d**) detachment (d-1) manufactured FITC-dextran-coated DNA microneedle (FITC: 100 mg/10 mL). The inset indicates the enlarged needle and confocal microscopy image (scale bar: (i-ii) 100 μm, (d-1) 200 μm).

To compare the diffusion of FITC with the vacuum timing of the DNA solution, vacuum was drawn at the start and termination of DNA solution application (in a semi-dry state). The total amount of DNA solution was 7.5 mL, and 2.5 mL of the DNA solution was initially poured into the mold loaded with FITC. For the first method, pouring of DNA solution was proceeded by 15 min of vacuum extraction (Figure 1(b-1)) and subsequent drying in an oven at 50 °C (Figure 1(c-1)). When the solution volume was reduced by drying in an oven to form the DNA needle structure, 1 mL of DNA solution was added five times, resulting in a total volume of 7.5 mL. 

**Figure 2:**
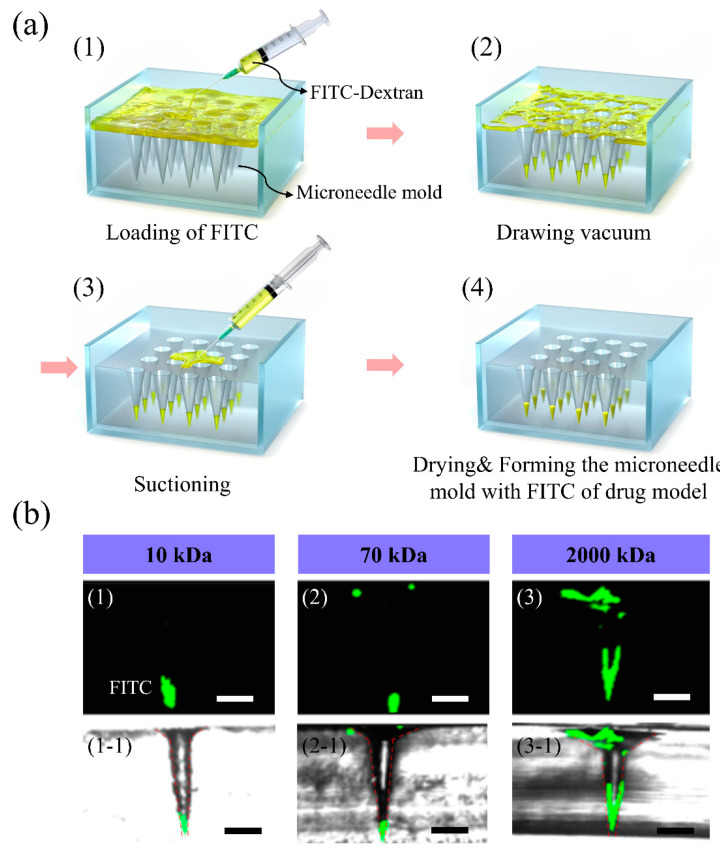
(**a**) Process of loading FITC-dextran of different molecular weights based on the dispensing and suction method. (1) Loading of the FITC-dextran solution into the prepared mold, (2) drawing vacuum, (3) suctioning, and (4) drying. (**b**) Merged optical and confocal microscopy images of FITC-loaded molds (1, 1-1) 10 kDa, (2, 2-1) 70 kDa, and (3, 3-1) 2000 kDa (scale bar: 200 μm).

**Figure 3:**
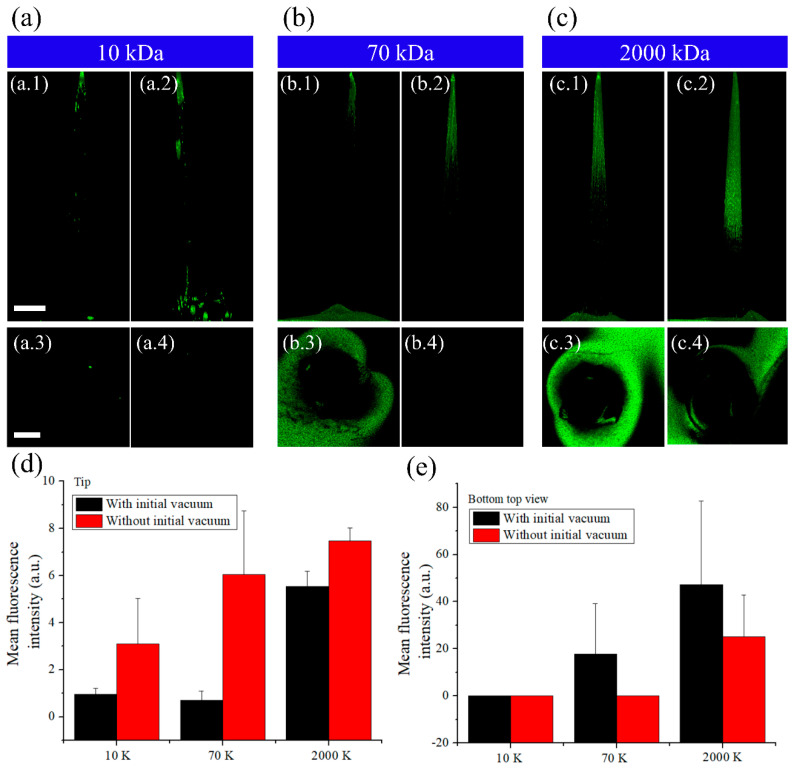
Confocal fluorescence images stacked along the z-axis of microneedles fabricated with (1,3) or without (2,4) initial vacuum, loaded with (**a**) 10 kDa FITC-dextran, (**b**) 70 kDa FITC-dextran, or (**c**) 2000 kDa FITC-dextran. The average fluorescence intensity of FITC-dextrans of different molecular weights (**d**) in the needle tip and (**e**) at the bottom (top view) (scale bar: 100 μm). The error bars represent mean ± standard deviation (*n* = 3).

The solution was dried in an oven until fully evaporated. The other method tested was the oven-drying process, which was done at 50 °C without the initial vacuum step and in the semi-dry state (Figure 1(b-2)). Finally, 1 mL of DNA solution was added, and when the total volume was 7.5 mL, vacuum was applied for 15 min to allow the solution to enter the tip mold loaded with FITC (Figure 1(c-2)) and dried in an oven at 50 °C (Figure 1(c-3)).

### 2.2. Device Analysis

#### 2.2.1. Confocal Microscopy

DNA MN-loaded FITC-labeled dextrans were characterized by confocal microscopy (LSM 510, Carl Zeiss, Germany). We equated all fluorescence control values for the control group to compare the fluorescence intensity while measuring. In the case of the FITC-loaded mold, the center of the hole portion (needle part) was cut to confirm fluorescence distribution in the mold. In the case of DNA-based needle-loaded FITC, the needle tip and lower portions were imaged by z-stacking from top view and projecting it to obtain a 3D needle image. For fluorescence data analysis, an ImageJ program was used, and the average green fluorescence intensity in the same area was compared. 

#### 2.2.2. Rheology Measurement for Viscosity Measurement

A rheometer (MCR 302, Anton Paar, Germany) was used to analyze rheological properties. A simple shear test was used to determine viscosity according to the concentration of the prepared DNA solution. The instrument comprised a cup and bob, with a cup diameter of 26 mm, height, 40 mm, and cylinder diameter, 20 mm. The conditions were measured at 30 °C, and the shear rate range was 0.05–1000 (1/s).

#### 2.2.3. Diffusion Simulation

The diffusion module of COMSOL Multiphysics software (Version 5.5, COMSOL Inc., *Stockholm,* Sweden) was utilized for the simulations. We solved the conservation form of the general convection and diffusion equations in COMSOL Multiphysics. The mold contained the solidified FITC-dextran and DNA solution. Diffusion equations were used to simulate solute diffusion in the solvent, and the solution was solved by applying interpolation as the viscosity of DNA solution varies with time.

## 3. Results and Discussion

FITC-dextran as a fluorescent dye can replace drugs for visualization, and it is suitable for drug modeling owing to its various possible molecular weights. Thus, FITC-dextran is widely applied in drug delivery experiments. Figure 1a shows the dispensing and suction method in the MN mold for drug tips. The syringe was used to control the drug volume in the dispensing and suction method (Figure 2((a-1),(a-3))). Importantly, the vacuum process ensures that the drug easily flows into the tip of the MN molds (see Figure 2(a-2)). To demonstrate the applicability of our method, FITC-dextran of different molecular weights was fabricated on the tips of MNs. Figure 2b shows the confocal microscope images of MN molds that were dispensed with FITC-dextran. Figure 2((b-1)–(b-3)) illustrates MN-tip loading with FITC-dextran of different molecular weights (10, 70, and 2000 kDa). The fluorescence height concentrated at the tip of the mold was distributed within 400 µm for high molecular weight (2000 kDa) FITC-dextran and within 200 µm for low molecular weight (10 and 70 kDa) FITC-dextrans. Therefore, for high molecular weight FITC-dextrans, it was confirmed that the drug was distributed in more areas with the dispensing and suction method. This explains why the low molecular weight FITC-dextran solutions were sucked more easily. Figure 2(b(1-1)–b(3-1)) shows the merged confocal and optical microscope images of the fabricated MN mold with constant amounts of FITC (cross section). The mold before drug loading was as follows (Supplementary Figure S1): the microtip height of the mold was 1000 μm and the distance between microtips was 2000 μm. When the drug was loaded into the mold using the dispensing and suction method, drug models by molecular weight in the mold were confirmed as shown in Supplementary Figure S1. The images are cross-sectional images of dextran of each molecular weight loaded with FITC and those when the amount of FITC increased. 

In the MN mold loaded with FITC, we tried to pour the DNA solution to fabricate the needle. Figure 1 illustrates the fabrication process of the FITC-loaded DNA MNs for retaining FITC in the tip as much as possible. Figure 1a shows the process of pouring the DNA solution onto the solidified FITC loaded in the mold. To reduce FITC diffusion, we performed adjustments to the sequence of vacuum steps (Figure 1(b-1,b-2)). The DNA MNs fabricated with initial vacuum application demonstrated active diffusion of the solidified FITC into the DNA solution during the drying process (enlarged Figure 1(b-i)). This diffusion phenomenon could affect the distribution of FITC at the tip of MNs, leading to drug loss. Therefore, we tried to use a semi-drying process for DNA solution in the first step to prevent the diffusion of FITC-dextran (Figure 1(b-2)). Figure 1(b-ii) shows the DNA solution facing the solidified FITC loaded in the mold using the semi-drying process, explaining that a substantial improvement can be achieved with this technique. This effect was due to the difficulty of FITC-dextran to diffuse into the DNA solution of relatively higher concentration poured into the MN mold. Notably, the implementation of initial vacuum influences the DNA concentration. This was also observed and explained by the simulations. In addition, the optical microscopy image of the system in the semi-dry state showed the status of the DNA solution (in the optical microscopy image of enlarged Figure 1(b-ii)). Figure 1c demonstrates the DNA solution drying process with and without initial vacuum, as illustrated in Figure 1((c-1)–(c-3)), respectively. In the two-step molding process by solvent evaporation, diffusion after drug loading should be considered for effective drug loading [29], which is generally controlled by the initial solvent concentration or solvent type. In the present study, the vacuum timing was changed to control the initial solvent concentration. The corresponding MN fluorescence images were compared and analyzed, as shown in Figure 3. The vacuum process was implemented when the DNA solution was in the semi-dry state, which can effectively prevent the diffusion of FITC-dextran. Figure 1(d-1) illustrates the fabricated DNA MNs with the FITC tip obtained using the semi-drying method (transparent yellow indicates the FITC tip).

To better evaluate the differences among MNs fabricated with FITC-dextran of different molecular weights and with different sequences of vacuum steps, confocal fluorescence images were obtained (Figure 3). Figure 3a–c shows DNA MNs, fabricated with and without the initial vacuum step, loaded with FITC-dextran of different molecular weights. The fluorescence images clearly demonstrated the anti-diffusion effect with/without initial vacuum. When drying without initial vacuum was performed to reduce the volume to half of the DNA solution, the DNA solution was unable to enter the tip portion, thereby forming an air pocket. Vacuum was then applied to draw the DNA solution into the tip, and the air pocket was removed. At this point, FITC contacts the DNA solution for the first time and FITC diffuses into the DNA solution. 

In this study, we focused on FITC as much (drug model) as possible within the MN, and therefore we compared the mean fluorescence intensity at the needle tip. We confirmed that FITC-dextran was more concentrated at the tip of MNs manufactured without the initial vacuum step. Figure 3d shows the fluorescence intensity at 50% of the needle tip height in tips manufactured with and without initial vacuum and loaded with FITC-dextran of different molecular weights. Without the initial vacuum step, more FITC is dispersed within 500-μm height of the tip. There was more than a four-fold increase in the distribution of FITC-dextran at the tip when loaded with 2000 kDa FITC-dextran than with 10 kDa FITC-dextran, and there was also a substantial difference in distribution between 10 and 70 kDa FITC-dextran. Figure 3e shows the distribution of fluorescent FITC-dextran in the lower region of the tip. Without the initial vacuum, a greater length of the MN was fluorescing with FITC (which faded towards the MN base). With the initial vacuum step, FITC spread rapidly, from the tip, reducing the amount of FITC at the needle tip. On the contrary, without the initial vacuum step, less amount of FITC diffused in the tip portion, and the needle base appeared to be relatively faded. This indicates that the diffusion of FITC at the tip is related to the dry state of the DNA solution and that it depends on the molecular weight of FITC-dextran. Fluorescence distribution in the DNA needle according to the molecular weight of FITC-dextran was different owing to the distribution of FITC loaded in the initial mold. The lower the molecular weight, the lower the distribution of the drug loaded using the suction method as it can be taken up with a syringe. With low molecular weight drugs, increasing the drug concentration can increase the drug load in the mold, without affecting the drug-loading efficiency (Supplementary Figures S1 and S2(d-1)), whereas in the mold loaded with high molecular weight drug, FITC was present near the base. These dextran polymers, with a high molecular weight, have long chains. Thus, FITC cannot be completely drawn owing to its weaker mobility. The higher the molecular weight, the higher the amount of FITC near the base, and the direction of FITC diffusion was also toward the base. Therefore, while loading a drug using the dispensing and suction method, FITC distribution in the MN tip varies with the concentration and molecular weight of the drug. In addition, it is important to consider the interface between FITC and DNA solution for a concentrated distribution of FITC at the needle tip in the solvent process of the needle manufacturing process.

**Figure 4:**
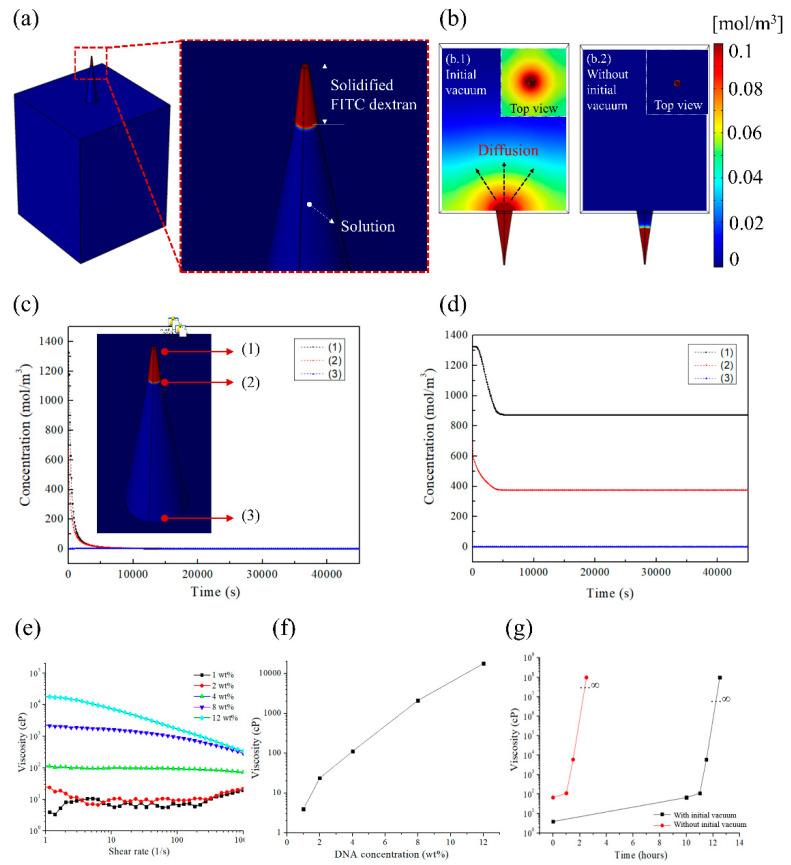
Diffusion simulation of the needle fabrication process. (**a**) Mold with FITC and DNA solution. The enlarged image shows the 200-μm high solidified FITC and 800-μm high DNA solution in the mold (time = 0 s, the interface before spread between the drug-loaded tip and top-up solution). (**b**) Needle diffusion after 45,000 s (b.1) with initial vacuum and (b.2) without initial vacuum. Graph of drug distribution by needle position over time (**c**) when initial vacuum was implemented; the inset image indicates positions 1, 2, and 3, respectively, and (**d**) when initial vacuum was not implemented. (**e**) Steady shear viscosity with shear rates for different DNA concentrations. (**f**) Viscosity values with the DNA concentration. (**g**) Relationship between DNA viscosity and actual drying time determined using an interpolation plot (when DNA and FITC are in contact).

To evaluate the differences in FITC distribution, the diffusion of drug in the MN mold solution was modeled. Diffusion numerical simulations were performed using the commercial FEM program COMSOL Multiphysics. To mimic the diffusion of solidified FITC using the DNA solution into the PDMS mold, unit cells, a part of the PDMS mold, were prepared for simulation (Figure 4). Among the diffusion modules, the transport of diluted species interface module was used, which interprets the delivery of the diluted solute in the solvent. As the solvent was water in polymer diffusion, the solute was the primary variable. The general convection and diffusion equation was used for the transport of diluted species module. When considering solute transport, concentration gradients induce diffusion, whereas convection contributes to solute flow during high fluid motion [31]. Therefore, simulations have been modeled for the combination of convection and diffusion.

(1) $$J_{i}=-D_{i}\nabla C_{i},$$

(2) $$\nabla \cdot J_{i}+u\cdot \nabla c_{i}=R_{i},$$

where *J_i_* is the flux of solute *i*, *D_i_* is the diffusion coefficient, and ▽*C_i_* is the concentration gradient of species *i*. The combination with the continuity equation for mass illustrates the Fick’s law. For diffusion simulation, each domain was largely divided into two zones. Zone 1 was with solidified FITC, where the initial concentration was applied (c1: 1326 (mol/m^3^)), whereas zone 2 was the solvent part, where the initial concentration of FITC was zero as FITC was present only in zone 1. The drug concentration in zone 1 was applied by calculating the actual loaded FITC concentration (a tip) (Supplementary Figure S2). Therefore, for diffusion simulation, the mold consists of the solidified FITC (solute) and solution (DNA molecular weight). During the experiments, the solidified FITC was filled to the 200 μm mark, approximately, and the bottom was filled with DNA solution (enlarged image of Figure 4a). In addition, heat was applied to dry the DNA solution on the solidified FITC-loaded PDMS. According to Wilke et al. [32], temperature and diffusion are inherently dependent. They follow the equation describing the diffusion coefficient of solute diffusion in a given solution. Solute diffusion in solution is influenced by various variables, such as molecular weight of solvent (*M*), temperature (*T*), viscosity (*η*), factor for solute solvent interaction (*ϕ*), and molar volumes of solute (*V*), as described using the following equation for the diffusion coefficient (*D*).

(3) $$D=\frac{7.4\cdot 10^{-8}{\left(\phi M\right)}^{0.5}T}{\eta V^{0.6}},$$

Therefore, the diffusion coefficient is a function of temperature, and the viscosity of the DNA solution is defined as a variable that changes as it dries. The diffusion coefficient was defined as a function of viscosity over time through the interpolation function. In the above formula [32], the temperature applied was 300 K (27 °C), and the temperature effect was corrected by adjusting *η*. The parameters applied for diffusion simulation are shown in Supplementary Table S1. Supplementary Figure S3 illustrates the mesh for diffusion simulation. The mesh was finely applied to the tip region (free tetrahedral).

Figure 4b shows the distribution of FITC via diffusion after the tip was treated with or without the initial vacuum step and shows the cross-sectional and top view images. Figure 4(b.1) represents fabrication with initial vacuum and Figure 4(b.2) represents fabrication without initial vacuum. As shown in Figure 4(b.1,b.2), drug distribution by diffusion can be observed (this trend is consistent with the data in Figure 1(b.1,b.2)). Figure 4c,d shows the changes in FITC concentration with time and tip position. Figure 4c shows the effect with initial vacuum, and FITC is rapidly diffused during the early stage. Figure 4d shows the effect without initial vacuum, and FITC is gradually diffused from the tip. These results show that FITC diffuses, solidifies, and stops diffusing. Therefore, the manipulation of DNA viscosity can prevent the diffusion of FITC and concentrate FITC at the tip. This experiment demonstrates the possibility of reducing FITC diffusion without altering the amount of DNA, and that FITC can be loaded at the tip. 

During the drying process, the DNA concentration, with or without the initial vacuum step, was confirmed by calculating the DNA solution volume and the evaporated solution poured into the mold. Figure 4e shows the rheometer measurement results predicting the rheological viscosity according to the DNA concentration. It shows the viscosity with shear rate for each concentration, and the predicted viscosity at each DNA concentration is replotted, as shown in Figure 4f. The predicted DNA viscosity at each time point when the DNA solution and the coagulated FITC are in contact is shown in Figure 4g. During the manufacturing process of the DNA-based MNs, the applied temperature (50 °C) was selected to fabricate the MNs. At this temperature, the DNA is stable [33]. The viscosity increased rapidly. The values shown in Figure 4g are essential for the diffusion simulation and were used in the simulation results above. 

**Figure 5:**
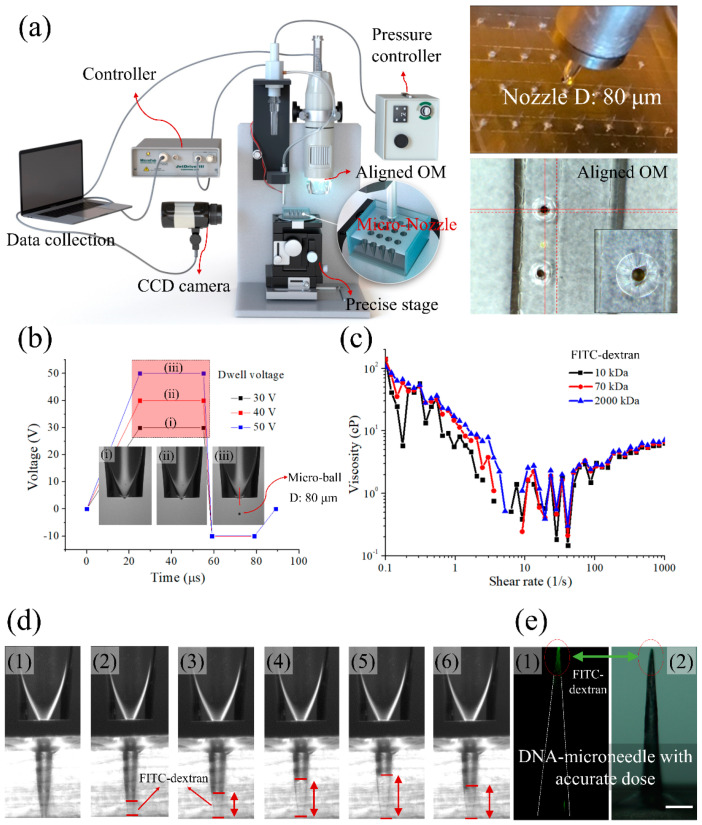
(**a**) Schematic diagram of the micro-jetting system for drug loading. Experimental images of the mold and nozzle, nozzle and mold for positioning, and top view image. The inset image shows the straightness of FITC solution loading into the mold. (**b**) Driving waveform conditions for stable loading of FITC at (b.i) 30 V, (b.ii) 40 V, and (b.iii) 50 V. (**c**) The effect of shear rate on steady shear viscosity of FITC-dextrans of different molecular weights (10 mg/10 mL), (**d**) micro-jetting of solution in the micro mold after (d.1) 0 drops, (d.2) 50 drops, (d.3) 100 drops, (d.4) 150 drops, (d.5) 200 drops, and (d.6) 1000 drops. (**e**) (e.1) Confocal microscopy image of the fabricated DNA needle-based FITC loaded by micro-jetting; (e.2) optical image of the microneedle (scale bar: 200 μm).

In addition to considering the diffusion phenomenon of the FITC-DNA solution occurring in the aforementioned two-step molding process, the micro-jetting method was applied to load precise amounts of the drug to the tip. This method has the advantage of being able to control drug loading accurately and quickly at the tip of the MN for future mass production. This system requires less than 100 μs to dispense a drop of a drug, enabling quantitative drug loading in a short period. In addition, in a MN drug delivery system, the size of the mold and the amount of drug manufactured vary depending on the daily dose required. Therefore, it is necessary to apply the drug precisely and quickly to only the tip for mass production. Based on the proposed method, an 80-μm nozzle was chosen. Figure 5a shows the schematic representation of the micro-jetting system and a photograph of the nozzle. The micro-jetting system is primarily composed of a pressure controller, optical microscope, micro-nozzle, and precise stage. As shown in Figure 5a, a CCD camera and an optical microscope were used to align the nozzle and MN mold. In addition, the FITC solution was loaded into the MN molds using a micro-nozzle. Notably, a micro-ball can be formed and successfully loaded into MN molds by adjusting the applied voltage. Moreover, the volume of the micro-ball (FITC solution) can be adjusted from nano to micrometer scales. Thus, it can achieve accurate fabrication based on the requirements of the drug to be delivered. 

The enlarged image in Figure 5a shows the nozzle and mold in the actual experiment. The nozzle used here was 80 μm in diameter for a macro-ball, and a nozzle suitable for a MN mold (bottom diameter: 200 μm) was also used. In addition, the mold and nozzle are aligned to properly load drugs from the nozzle (magnified top view). Figure 5b shows the anode trapezoidal waveform, which was the driving waveform for micro-jetting. It is difficult for the FITC solution to drop from the nozzle when the dwell voltage is low (Figure 5((b.i),(b.ii))). Therefore, appropriate voltage regulation is essential. The viscosity of the solution is also an important aspect of jetting because high viscosity can block the nozzle. Figure 5c shows the measurement of viscosity at various shear rates for 10 mg/mL FITC solution of different FITC-dextran molecular weights. It was found that the viscosity of the FITC-dextran solution used for drug modeling was suitable for micro-jetting. Figure 5d shows the injection of FITC solution into the mold. Figure 5((d.1)–(d.6)) shows that when the drug is continuously injected into the mold, the FITC solution is filled into the mold, and when the solution dries in the mold, the height appears to be slightly lowered. A total 1000 drops was loaded and a fluorescence image of the needle is shown in Figure 5e. It was confirmed that there was a small amount of well-positioned FITC at the tip.

## 4. Conclusions

In this study, we developed an effective dispensing method for fabricating DNA MNs with drug-containing tips based on the initial vacuum timing. The diffusion of FITC-tips can be well inhibited by semi-drying, thereby loading FITC only to MNs, which can reduce the loss of drug. FITC-dextran of different molecular weights was fabricated on the tips of DNA MNs to demonstrate the applicability of our method. Numerical simulation of diffusion explained the importance of the anti-diffusion phenomenon, which is based on the DNA solution facing the solidified drug model. The distribution of the loaded drug was analyzed with and without an initial vacuum step. Finally, a micro-jetting system was developed to load an exact amount of drug into the MN. Voltage was adjusted to maintain the straightness of the stable drug drop and confirm drug loading into the mold, while the drug volume can be adjusted from nano to micro volumes. We achieved a small amount of FITC loaded at the tip of DNA MNs with 1000 drops. We believe that this new anti-diffusion technique and precise micro-jetting method will be beneficial for MN fabrication in multiple ways, including the skin penetration depth, exact drug loading, and prevention of drug loss at the tip during drug delivery. This method improves drug distribution and minimizes drug loss in the MN drug delivery system. In the future, we will complement this automation system with physical enhancers, such as electricity and ultrasound, for the large-scale manufacture of patches and drug delivery systems. 

## Supplementary Materials

The following are available online at https://www.mdpi.com/1999-4923/12/10/954/s1, Figure S1: Confocal fluorescence and merged images of mold cross-sections before and after drug loading, Figure S2. Geometric structure of domains, Figure S3. Mesh images, Table S1: Material parameters employed for domains 1 and 2 in diffusion simulation.
