# Supplementary material for: Effective Dispensing Methods for Loading Drugs Only to the Tip of DNA Microneedles
Source: Pharmaceutics. 2020 Oct 10;12(10):954. doi: 10.3390/pharmaceutics12100954 (PMC7599544; doi:10.3390/pharmaceutics12100954)
Supplement: Supplementary file 1 [file pharmaceutics-12-00954-s001.pdf]

pharmaceutics

Supplementary Materials: Effective Dispensing Methods for Loading Drugs Only to the Tip of DNA Microneedles

Moonjeong Bok, Zhi-Jun Zhao, Soon Hyoung Hwang, Hyeok-Joong Kang, Sohee Jeon, Jiwoo Ko, Jiwon Jeong, Young Seok Song, Eunju Lim and Jun-Ho Jeong

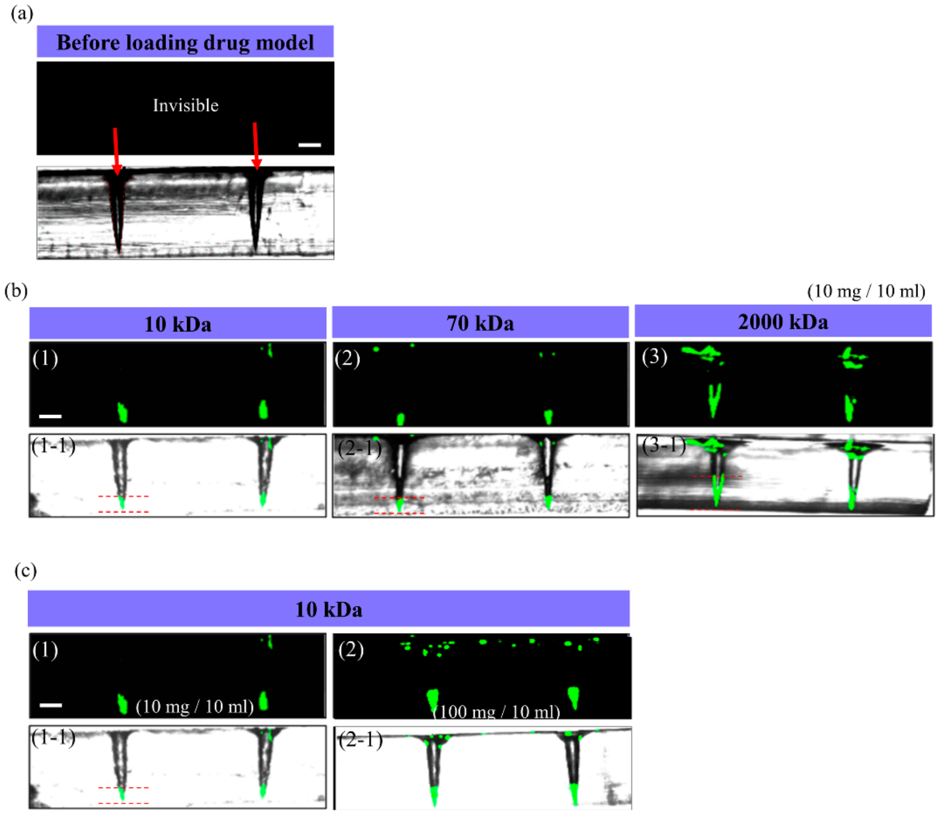

Figure S1. Confocal fluorescence and merged images of mold cross sections before and after drug loading. (**a**) Before loading; (**b**-1) 10 kDa, (**b**-2) 70 kDa, and (**b**-3) 2000 kDa with 10 mg/10 mL; (**c**-1) 10 mg/10 mL, and (**c**-2) 100 mg/10 mL with 10 kDa (scale bar: 200 μm).

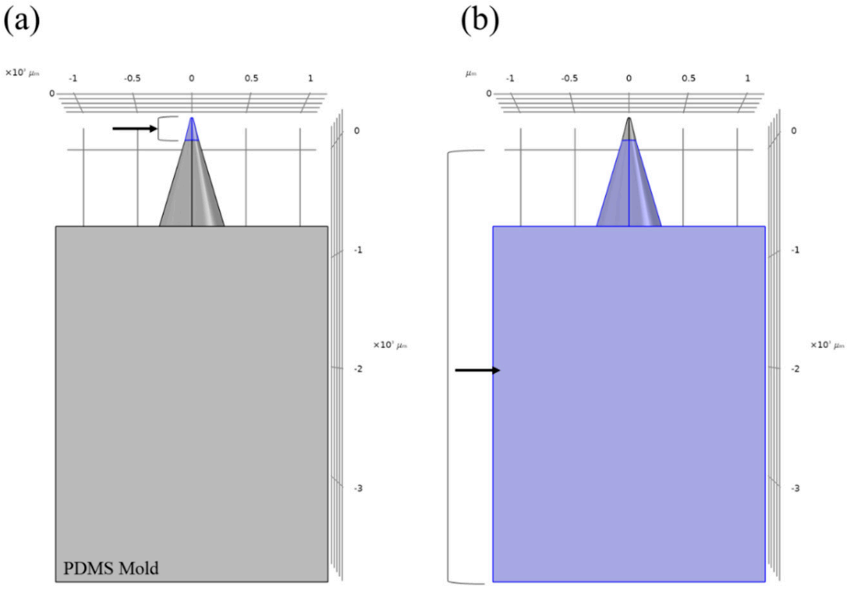

Figure S2. Geometric structure of domains. (**a**) Domain 1; (**b**) Domain 2.

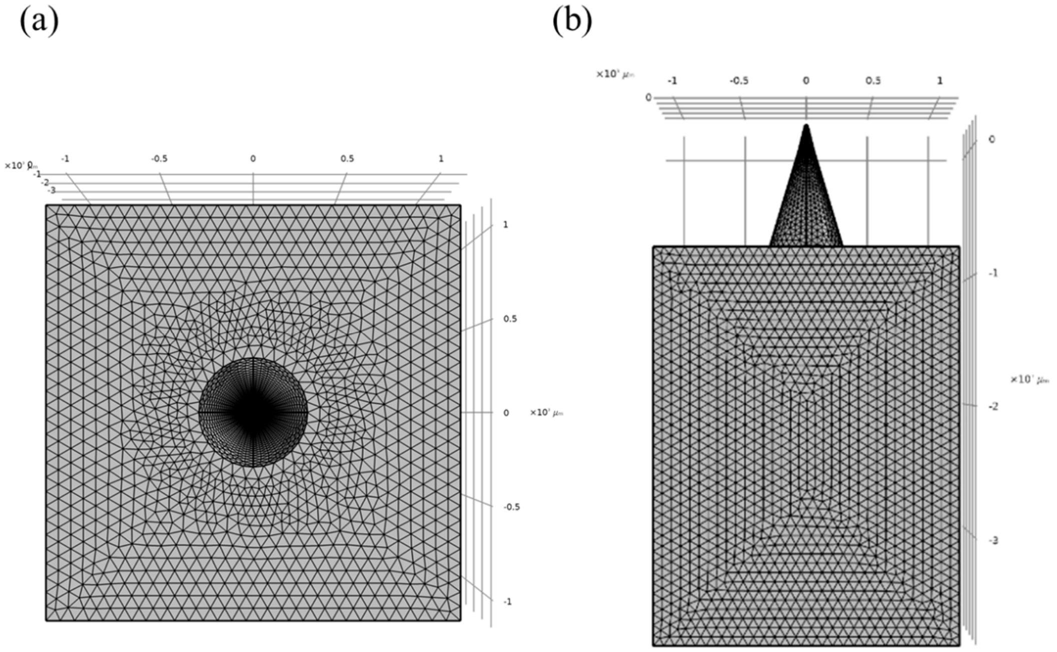

Figure S3. Mesh images. (**a**) Top view; (**b**) cross section.

Table S1. Material parameters employed for domains 1 and 2 in diffusion simulation.

	**Domain 1**	**Domain 2**
Concentration (c, mol/m^3)	1326	0
Molar volume of solute (v, cm^3/mol)	7.54×10^2	
Molecular weight (M, g/mol)	10^4	8×10^5
Temperature (T, K)	300	300
